# Rutting and rambling: Movement characteristics reveal partial migration in adult male white‐tailed deer at a latitude void of chronic and severe environmental fluctuations

**DOI:** 10.1002/ece3.10875

**Published:** 2024-02-13

**Authors:** Luke Resop, Stephen Demarais, Bronson K. Strickland, William T. McKinley, Garrett Street

**Affiliations:** ^1^ Department of Wildlife, Fisheries, and Aquaculture Mississippi State University Mississippi State Mississippi USA; ^2^ Mississippi Department of Wildlife Fisheries, and Parks Jackson Mississippi USA

**Keywords:** excursions, home range, migration, movement strategy, partial migration, white‐tailed deer

## Abstract

White‐tailed deer (*Odocoileus virginianus*) are generally considered a home‐ranging species, although northern populations may migrate between summer and winter ranges to balance resource requirements with environmental stressors. We evaluated annual home range characteristics of adult bucks (*n* = 30) fitted with GPS collars from 2017 to 2021 in central Mississippi with time series segmentation and Kernel Density Estimation (KDE) to determine if individuals employed varying movement strategies. We found 67% of bucks displayed a “sedentary” strategy characterized by a single KDE home range polygon with a mean size of 361 ha. The remaining 33% of bucks employed a “mobile” strategy characterized by multiple home range segments with a mean size of 6530 ha. Sedentary bucks went on an average of 5.9 excursions annually while mobile bucks went on 0.8. Excursion timing for both strategies peaked in breeding season and early spring. Mobile buck home ranges were separated by a mean distance of 7.1 km and mean duration in one home range segment before traveling to another was 78 days. Our study provides the first evidence that partial migration may apply to a larger proportion of lower‐latitude deer populations than originally thought, though the environmental justification for this partial migration is not clear.

## Graphical abstract

Some white‐tailed deer populations are migratory in locations with severe and chronic seasonal resource limitations, but biological justification for such behaviors is lacking at lower latitudes. We document migratory behavior termed the "mobile" movement strategy in 33% of adult male deer in a low‐latitude system void of such environmental pressures. Mobile deer had multiple home range segments separated by an average of 7 km and migrated at all times of year.

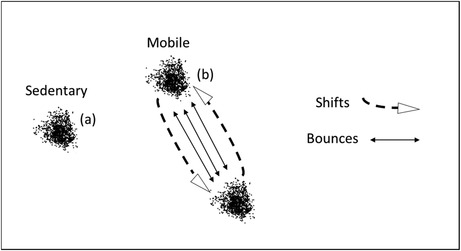

## 1 INTRODUCTION

Animals move in part because resources are not homogeneously distributed across the landscape (Bell, 2012; Nathan et al., 2008). Resources like food, cover, and breeding opportunities are often spatially and temporally constrained (White, 2008). Both species‐level and individual‐level requirements for these resources dictate the movement scale necessary to meet life history demands including survival and reproduction (Ovaska, 1988; Pomilla & Rosenbaum, 2005). For example, Griffon vulture (*Gyps fulvus*) movements vary with bioclimatic factors like food availability and flight conditions but are highly variable among individuals even when resources are predictable on the landscape (Monsarrat et al., 2013).

Within a species, individuals often exhibit varying strategies to navigate their environment and procure resources from it (Bell, 2012). Migration is one such movement behavior that populations may exhibit at varying degrees. Migration can be defined as cyclic seasonal movements allowing individuals to capitalize on spatially and temporally heterogeneous resources (Dingle & Drake, 2007). One example of a biological impetus for migration in high‐latitude terrestrial animal populations is when thermal refuge during extreme low temperatures and deep snowfall is spatially distinct from resources the animals require during the reproductive season (Sabine et al., 2002). This spatiotemporal mismatch incites seasonal migration between areas conferring reproductive success and those conferring winter survival. Although specific environmental contexts vary, the general ecological principles that drive migration apply to animals like the caribou (*Rangifer tarandus*) of North America (Nicholson et al., 2016), wildebeest (*Connochaetes taurinus*) in the Serengeti of Africa (Holdo et al., 2009), and Magellanic penguins (*Spheniscus magellanicus*) of the southeastern Pacific (Skewgar et al., 2014), each moving between seasonal resources to optimize survival and reproductive success.

Partial migration is a phenomenon describing populations in which some individuals migrate and others are resident (Chapman et al., 2011) and is documented in invertebrates (Hansson & Hylander, 2009), birds (Boyle, 2008), and mammals (Berg et al., 2019). Partially migratory populations are thought to arise from inter‐annual or inter‐seasonal unpredictability in resource availability, thereby conferring advantages to different strategies at different times. Although partial migration often occurs in environments with environmental stressors similar to those inciting full migration, such stressors are spatially or temporally inconsistent enough to justify different strategies within the population (Chapman et al., 2011). Environmental factors that may influence these movements include forage availability (Jahn et al., 2010), thermal stress (Monteith et al., 2011), disturbances like flooding (Abernathy et al., 2019), and biological variables like competition and predation risk (Chapman et al., 2011).

White‐tailed deer (hereafter deer; *Odocoileus virginianus*) are an ideal species in which to evaluate migratory behaviors because of their broad geographic range which covers many ecological contexts (Heffelfinger, 2011). Deer exhibit wide variations in movement tendencies, both at inter‐ and intra‐population scales (D'Angelo et al., 2005; Webb et al., 2007). Short‐distance movements include those associated with foraging (Etzenhouser et al., 1998), seeking cover (Ager et al., 2003), predator avoidance (Sweeney et al., 1971), and mate searching (Foley et al., 2015), while long‐distance movements are generally infrequent and include dispersal (McCoy et al., 2005), excursions (Karns et al., 2011), and migration (Sabine et al., 2002). Some high‐latitude deer populations are considered partially migratory (Henderson Jr. et al., 2018) as a portion of the population moves from summer range to wintering grounds associated with forage resources, dense evergreen tree cover for thermal protection, and easier movement through deep snow (Sabine et al., 2002; Verme, 1973). However, there is little obvious biological justification for partial migration in lower‐latitude deer populations of the southeastern United States because winter environmental conditions are less severe than at higher latitudes. Perhaps as a result, there is scant documentation of partial migration in deer populations at lower latitudes (Mott, 1981).

Our goal was to better understand deer movement and home range characteristics at a latitude void of chronic and severe winter weather. Pursuant to this, we sought to determine the proportion of adult male deer that display alternative movement‐based behaviors by evaluating net‐squared displacement (NSD) and home range characteristics (Bocci et al., 2010; Gaudry et al., 2015). Operating under the hypothesis that partial migration emerges in deer due to seasonal resource constraints (Nelson, 1998; Van Deelen et al., 1998), we predicted the minority of animals in our study would display migratory behaviors due to our study sites' relatively mild and stable bioclimatic conditions (Sabine et al., 2002). In addition to characterizing home ranging and migration patterns of our study populations, we sought to quantify other behaviors such as excursion timing, distance, and frequency. We predicted migratory individuals would embark on fewer excursions than non‐migratory individuals due to the energetic costs of long‐distance movements and the animals' limited capacity to support such movements (Shepard et al., 2013). A clearer picture of the characteristics and frequency of deer movement strategies could inform managing diseases (Grear et al., 2006; Mateus‐Pinilla et al., 2013), deer populations (Webb et al., 2007), and hunter perspectives (Mitterling et al., 2021).

## 2 STUDY AREA

**FIGURE 1:**
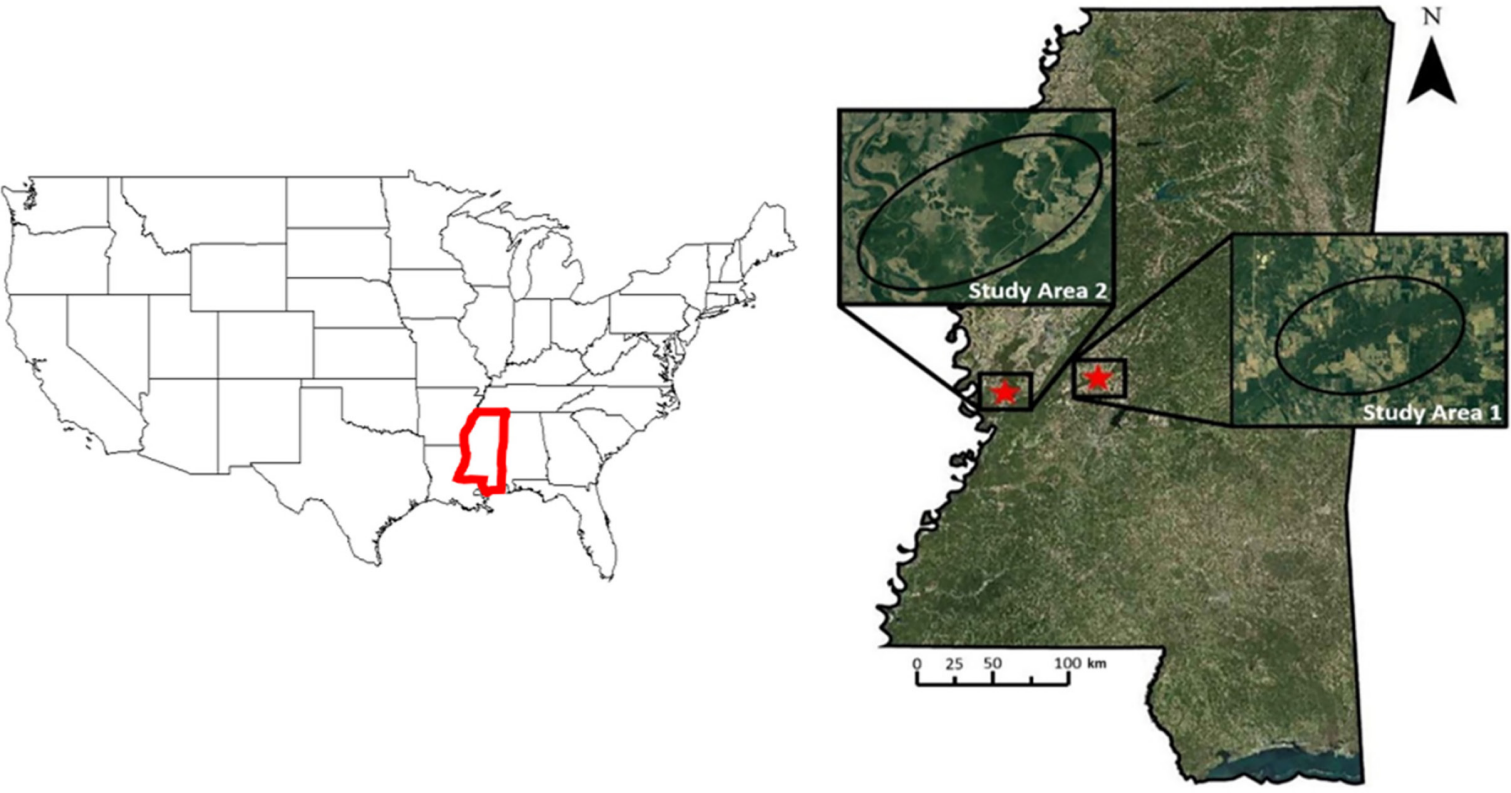
Study area 1 (approximately 20,000 ha) along the Big Black River and study area 2 (approximately 15,000 ha) along the Mississippi River in central Mississippi, USA.

Our study was conducted from 2017 to 2021 in central Mississippi, USA, on two separate study sites (Figure 1). The first site is along the Big Black River in Madison and Yazoo counties on approximately 20,000 ha of private land. The second is along the Mississippi River in Issaquena and Humphreys counties on approximately 15,000 ha of public and private land. These regions of Mississippi are characterized as a subtropical humid climate, with long, hot summers and short, mild winters (Mississippi Climate, 2021). Mean daily high temperature for this region in summer (June–August) is 33°C and 14°C in winter (December–February). Mean annual precipitation is 137 cm with 1.5 cm of snowfall. The study areas are located in an alluvial valley with flat topography.

Study area one land cover is characterized by two major groups: forest and agriculture. Coniferous forests comprise approximately 3000 ha (15%) of the study area and are predominantly loblolly pine (*Pinus taeda)* plantations (Henderson et al., 2020). Upland mixed deciduous forests comprise approximately 3400 ha (17%). Commercial agriculture (cotton, corn, soybeans, and peanuts) is the second most common land cover, making up 6000 ha (30%) of the study area. Study area two land cover is predominantly bottomland hardwood forest and commercial agriculture. Bottomland hardwoods comprise approximately 8200 ha (55%) and commercial agriculture another 3700 ha (25%; Jones et al., 2008). Both study areas are prone to flooding, but study area two experiences more frequent severe floods given its proximity to the Mississippi River. Peak breeding for deer in study area one occurs from 25 December through 7 January and 20 December through 2 January in study area two. The mean parturition date for study area one is 20 July and 14 July for study area two (Mississippi Department of Wildlife Fisheries and Parks, unpublished data). Since both study areas have considerable coverage of warm‐season agricultural crops, summer nutritional availability to support reproductive demands for does and antler growth for bucks is high relative to other parts of the state (Jones et al., 2008).

## 3 METHODS

### 3.1 Animal capture

We used dart rifles (Pneu‐Dart, Inc., Williamsport, Pennsylvania) to capture male deer ≥1.5 years old during September 2016 to August 2018 in study area one and December 2020 to November 2021 in study area two. We anesthetized deer using a solution of butorphanol, azaperone, and medetomidine (BAM; Zoopham, Windsor, Colorado, USA; Mich et al., 2008). We set dosages at 2.7 cc of BAM, with additional BAM administered if necessary. We attached GPS collars (Iridium Track M 3D, Lotek Wireless, Inc., Newmarket, Ontario, Canada) and uniquely numbered plastic and metal ear tags (Allflex U.S. Inc.) to each deer. Fix rates varied seasonally, but we used only fixes at four‐hour intervals for consistency across seasons. While anesthetized, we estimated age (Severinghaus, 1949), measured body mass, neck diameter, and Boone and Crockett antler score. We injected each deer subcutaneously with 3 cc of Nuflor (Merck Animal Health, AN Boxmeer, Netherlands) per 45 kg of body mass to prohibit possible infections. Reversal of BAM was performed using atipamezole at twice the total amount of BAM coupled with constant 0.5 cc of naltrexone. All capture methods were approved by the Mississippi State University Institutional Animal Care and Use Committee (Protocol #16–621 and 20–339).

### 3.2 Home range classification

We restricted analyses to deer with ≥11 months of consecutive GPS data (*n = 30 animals*) to ensure each deer had adequate time to display alternative movement strategies. Some individuals had full two‐year data sets producing a total of 44 buck‐years. We calculated NSD for each annual data set using the adehabitatLT package in program R (Calenge, 2006). Deer were visually separated into two classes prior to further analysis based on NSD plots and raw GPS points (Cagnacci et al., 2011; Mysterud et al., 2011). Data from deer with NSD plots and location clusters indicating spatially distinct areas of use were further analyzed using Lavielle time‐series segmentation in the adehabitatLT package. Lavielle segmentation is an algorithmic approach to segment time‐series data based on changes in mean and/or variance of a dependent variable over time (for our purpose, NSD; Lavielle, 2005). We do not report Lavielle segmentation for individuals without apparent spatial clustering because the procedure would segment changes in NSD that were not representative of the spatial scale at which our objective was directed. Even if we segmented all individuals' data, segments of animals with apparent spatial clustering would not be comparable to those without.

We conducted annual home range analyses from two perspectives: the utilization distribution and the occurrence distribution. These differ conceptually in that the utilization distribution measures the area within which the animal could conceivably occur over the entirety of its movements (including movements before and after observation), whereas the occurrence distribution measures where the animal could conceivably occur only during the period of observation, and each perspective must be addressed differently (Fleming et al., 2015). We estimated the utilization distribution using classical Kernel Density Estimation (KDE), and the occurrence distribution using Brownian Bridge Movement Models (BBMM) in package *adehabitatHR*. For both distribution types we estimated both the 95% and 50% isopleths to reflect broad and core space use trends, respectively. We chose the ad hoc smoothing method for KDE because it is less liberal than the *reference* method and less conservative than the *least‐squares cross validation* method, thus providing isopleths with intermediate smoothing (Rodgers & Kie, 2010; Worton, 1995). For the BBMMs, we estimated the first smoothing parameter related to movement speed for each buck with the function *liker* and used 20 as the second smoothing parameter value (related to imprecision of relocation in meters) for all bucks. Although only KDE is included in our formal analyses, we provide BBMM values as a reference for the reader since BBMM is a more conservative estimator (less empty space between GPS points and isopleth) than KDE for animals displaying migratory movements (Fleming et al., 2015).

**FIGURE 2:**
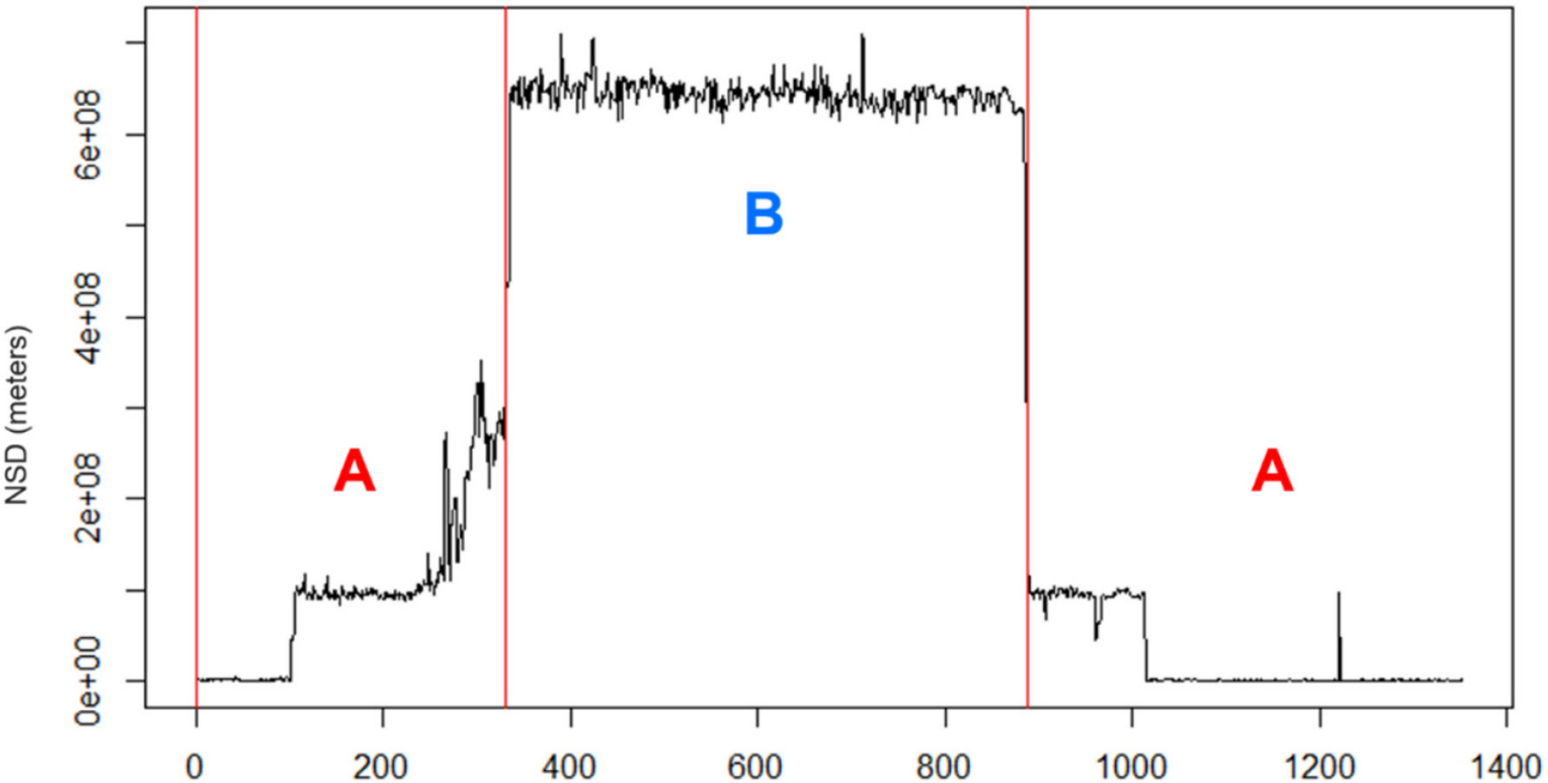
Example Net Squared Displacement plot from GPS locations. Lavielle segments are distinguished by vertical red lines at which a shift between home range segments occurs. For traditional Kernel Density Estimation (KDE) and Brownian Bridge Movement Models, all GPS data were used to estimate 50% and 95% utilization distributions (UD). In our segmented approach to KDE, all spatially overlapping segments labeled A were extracted, grouped, and used to create a UD. We applied the same process to segments labeled B, resulting in two home ranges for each individual.

We applied a third home range estimation method only for bucks with spatially distinct relocation clusters to minimize the amount of negative space between isopleth boundaries and GPS points. After extracting all relocations within a Lavielle segment, we combined data from spatially overlapping segments and estimated 95% and 50% KDEs on the segmented data (Figure 2), resulting in two separate sets of home ranges for each individual.

We used the non‐segmented KDE method for all analyses and reporting except for calculating the distance between home range segments, for which we used the segmented method. Segmented KDE and BBMM estimates are otherwise for net space use comparisons only (see Table A1).

**FIGURE 3:**
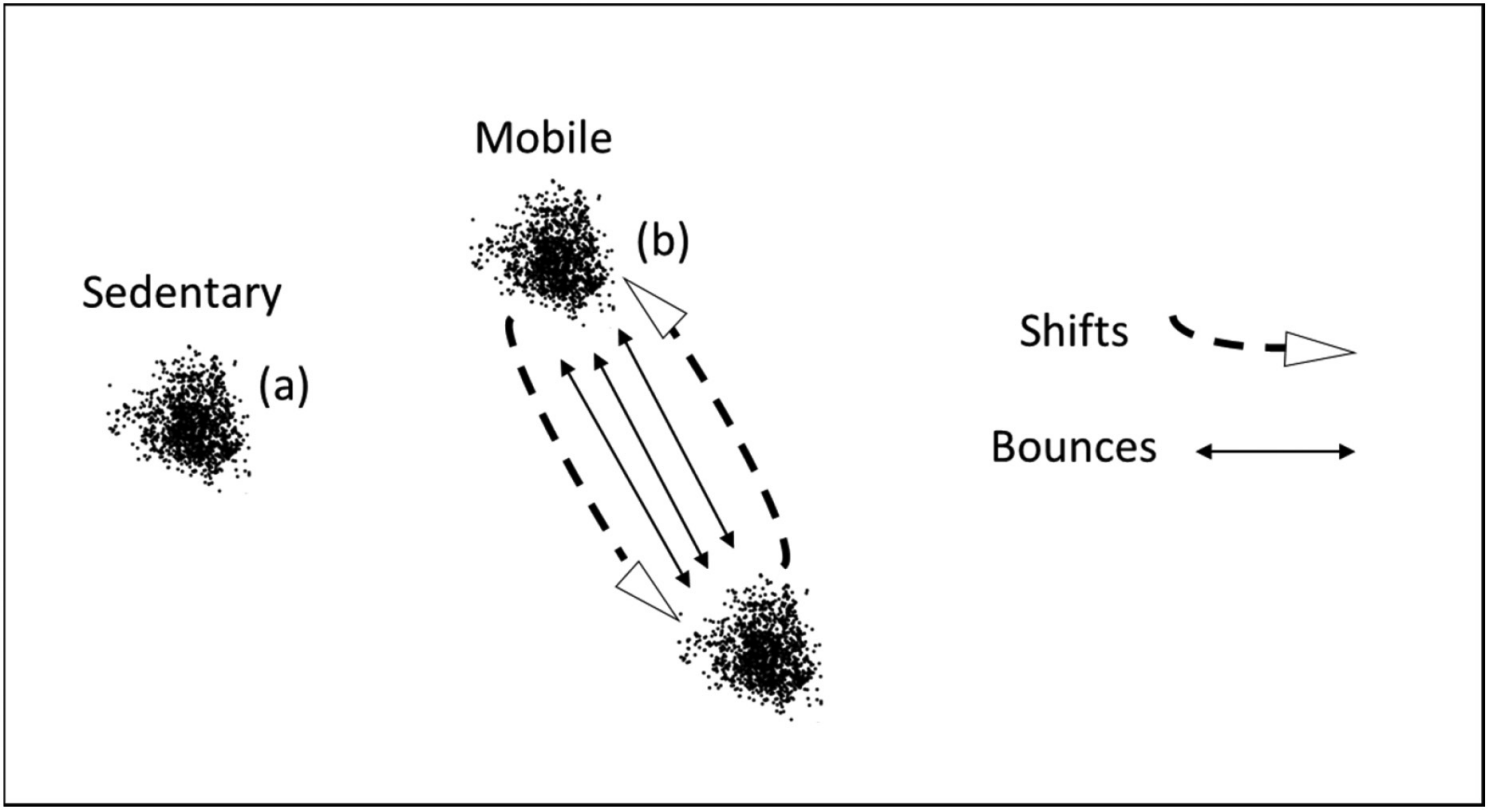
Relocation cluster patterns and respective strategy classifications for GPS collared bucks. Sedentary bucks (a) have a single cluster of relocations and mobile bucks and (b) have spatially discrete clustering. Within the mobile strategy, shifts represent less‐frequent trips between home range segments after which a buck remains in the home range segment for an extended duration while bounces are rapid intra‐Lavielle segment trips back and forth to his other home range segment.

We classified bucks as either “mobile strategy” or “sedentary strategy” based on relocation clustering. Those with spatially separated clusters of relocations and dramatic cyclic changes in NSD over time were considered mobile, and those without such characteristics were sedentary (Figure 3). Within the mobile classification, we classified rapid intra‐Lavielle segment trips between home range segments as “bouncing” movements, distinguished by short‐duration spikes in NSD. We only counted round trips to and from the other home range segment as bounces (Figure 3b). We also distinguished shifts between home range segments. Opposed to bounces, shifts are one‐way trips and are strictly indicated by the segments created by Lavielle algorithm (Figure 2) and are indicative of longer‐duration visits to home range segments. We consider bouncing and shifting as sub‐strategies since not all bucks display bouncing movements, but our sample size of each sub‐strategy was not large enough to justify statistical analyses comparing the two.

### 3.3 Analysis

We calculated home range size and distance metrics in ArcMap v.10.7 (ESRI, Environmental Systems Research Institute: Redlands, California, USA). Home range size for traditional KDE and BBMM methods was simply the total isopleth area. Since the segmented KDE method produced two home range isopleths, we calculated home range size as the total non‐overlapping isopleth area. Excursions were defined as any movement ≥0.5 km from the 95% KDE isopleth (Jacobsen et al., 2020; Karns et al., 2011; Kolodzinski et al., 2010). Excursions look similar to bounces between home ranges on an NSD plot, so we differentiated them in ArcMap prior to classification. Duration in segment for mobile bucks was calculated as the difference between segment start and end date produced in the Lavielle segmentation process, but we excluded each buck's first and last segments from consideration because of unknown first segment start date and last segment end date. Distance between home range segments was measured between 50% core area centroids from segmented KDE utilization distributions. We opted to use the segmented approach for this step to ensure all mobile bucks had two core areas to measure between. With the traditional unsegmented KDE approach, some mobile bucks had a single core area, making measuring between core areas impossible.

We used linear models in the *nlme* package to test differences between strategies. Analyses include the effect of strategy (independent variable) on home range size, duration in home range segment, distance between segments, and excursion duration and distance (dependent variables). We also tested the effect of buck age (independent variable; estimated to a specific age class; e.g. 2.5 years old) on strategy (dependent variable) to determine if strategy changed as bucks matured. We included buck ID as a random effect on the intercept in all models with observations of the same buck for multiple years to account for the variation in an individual buck's movement patterns from year to year. We analyzed frequency distribution relationships for timing of home range shifts, bounces, and excursions with chi‐square goodness‐of‐fit tests and strategy effect on excursion timing with a paired t‐test, both in the *stats* package. Standard deviation follows reported averages not part of a model output.

## 4 RESULTS

**FIGURE 4:**
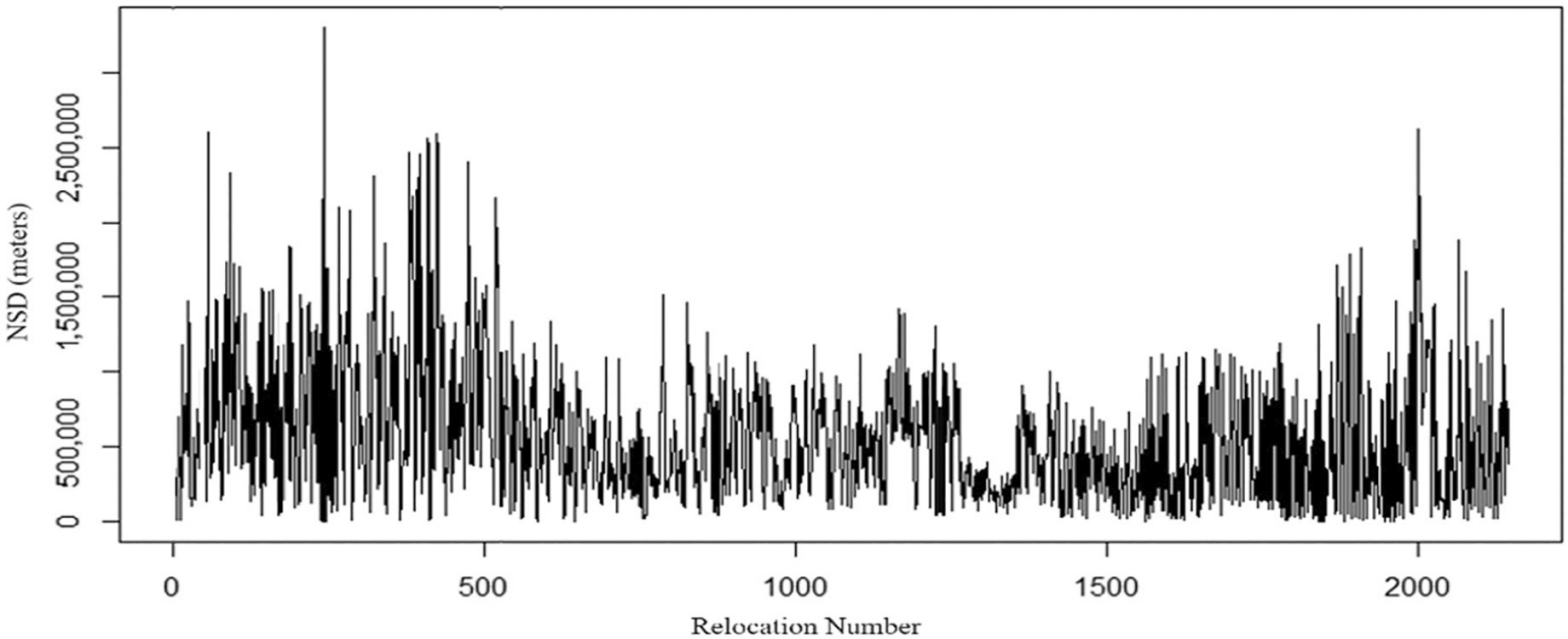
Buck #13 has a “sedentary” strategy characterized by relatively stable and non‐cyclic changes in NSD over time. Buck #13's GPS locations in this plot were taken during 2017–2018 in the Big Black River Region of Mississippi, USA.

Bucks with a sedentary strategy comprised 67% (*n* = 20) of our sample and had a mean home range size of 361 ha (*F*_1,42_ = 5.35, *p* = .028) when tested against mobile bucks. Sedentary bucks had single home range isopleths (Figure 3a) and stable, non‐cyclic changes in NSD over time (Figure 4).

**FIGURE 5:**
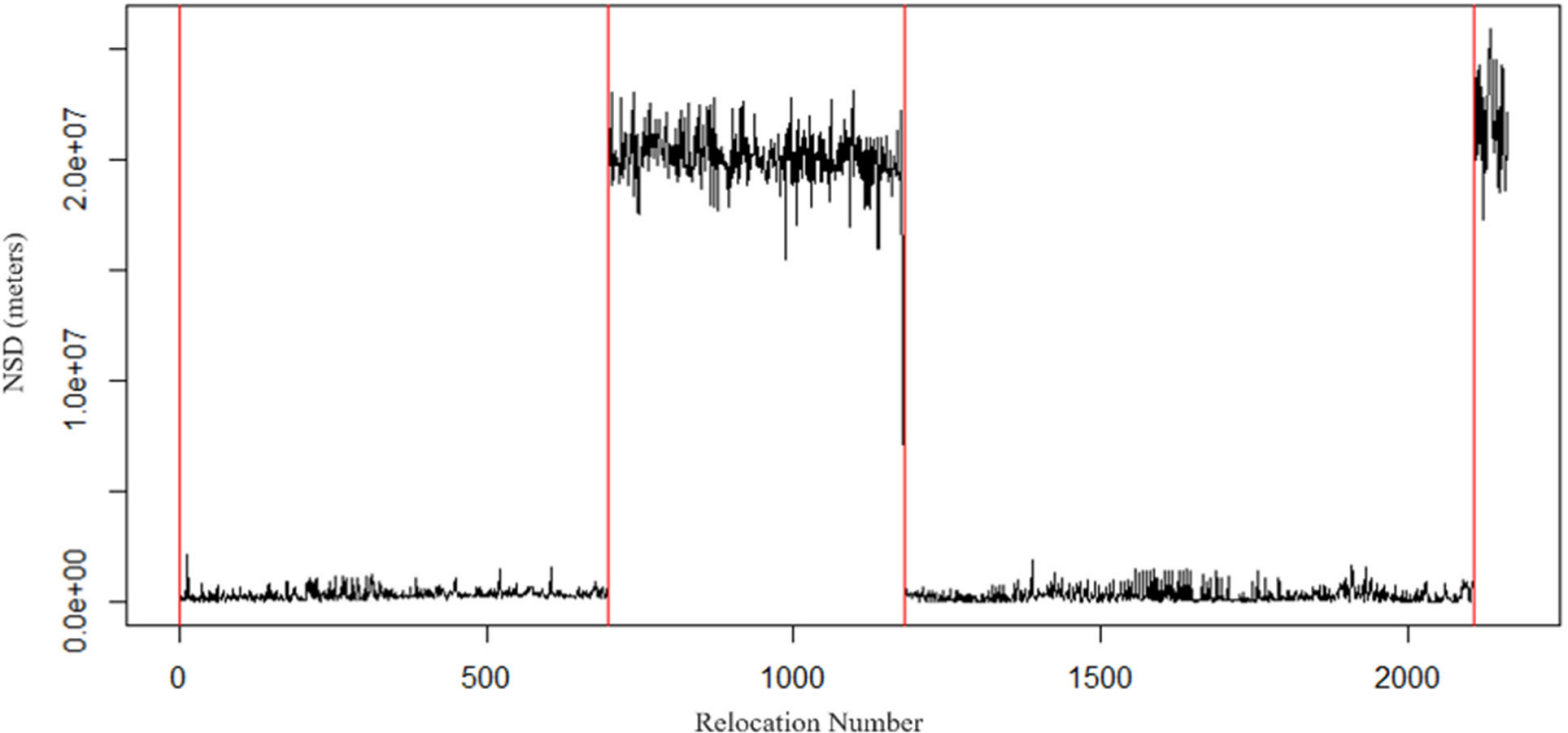
Buck #297's year one data is an example of a “mobile” strategy characterized by dramatic and cyclic shifts in NSD over time. Lavielle segments are distinguished by vertical red lines. Buck #297's GPS locations in this plot were taken during 2017–2018 in the Big Black River Region of Mississippi, USA.

Mobile strategy bucks comprised the remaining 33% (*n* = 10) of our sample and had a mean home range size of 6530 ha (*F*_1,42_ = 8.99, *p* = .006). Bucks #27 and #140 have outlier home range sizes at 20,070 ha and 36,559 ha, respectively. When these bucks are removed from the analysis, the mean home range size for mobile bucks is 1096 ha (*F*_1,40_ = 43.13, *p* ≤ .001). Mobile bucks had spatially separated clusters of relocations (Figure 3b) and dramatic, cyclic changes in NSD over time (Figure 5).

**FIGURE 6:**
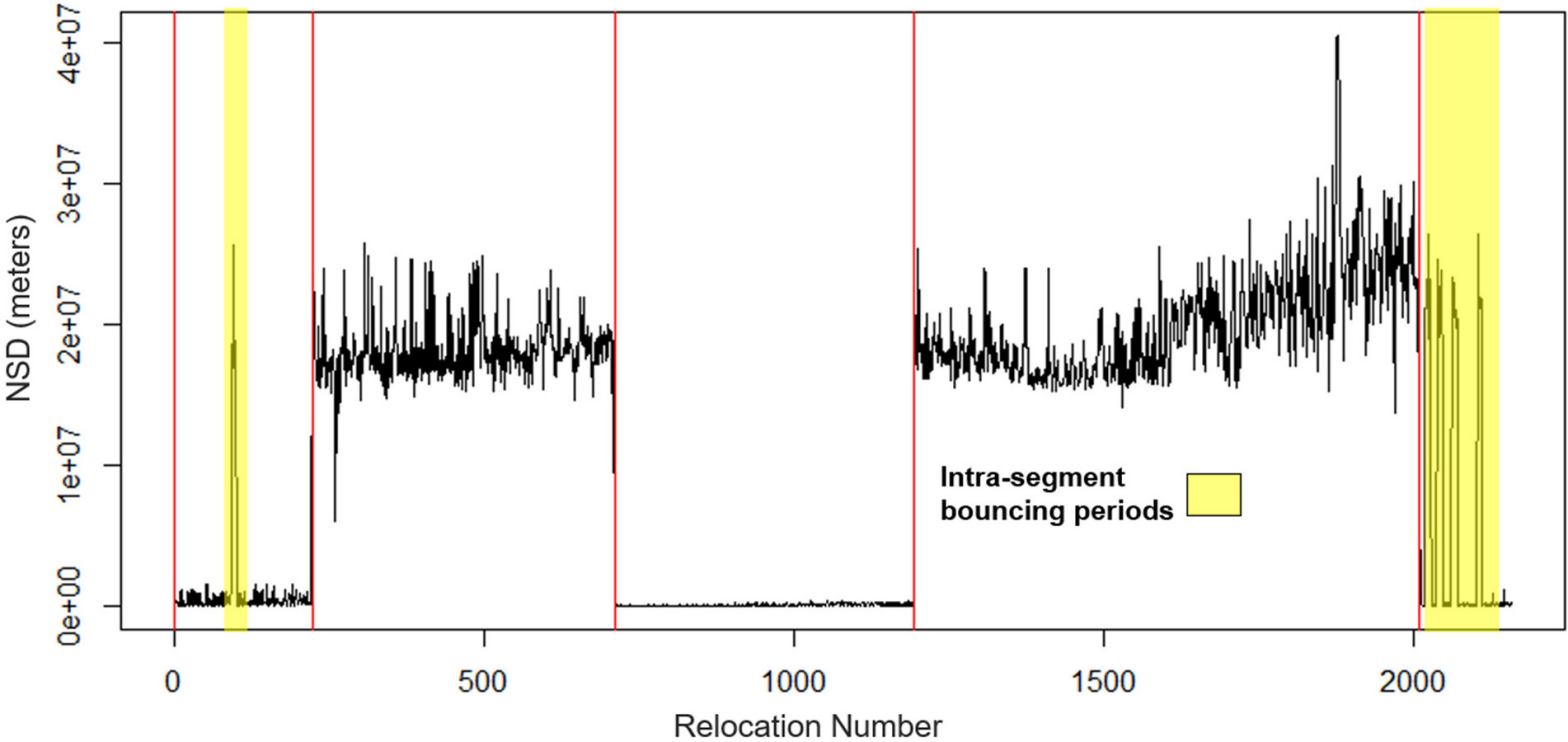
Buck #297's year two data contain “bouncing” behaviors, characterized by dramatic and cyclic shifts in NSD over time within Lavielle segments distinguished by vertical red lines. Bounces differ from excursions in that bounces are rapid trips back and forth between home range segments, whereas excursions are short‐duration trips to a location outside the home range boundary. Buck #297's GPS locations in this plot were taken during 2018–2019 in the Big Black River Region of Mississippi, USA.

Of mobile bucks, seven displayed bouncing behaviors distinguished by rapid intra‐Lavielle segment trips back and forth between home range segments (Figure 6).

**FIGURE 7:**
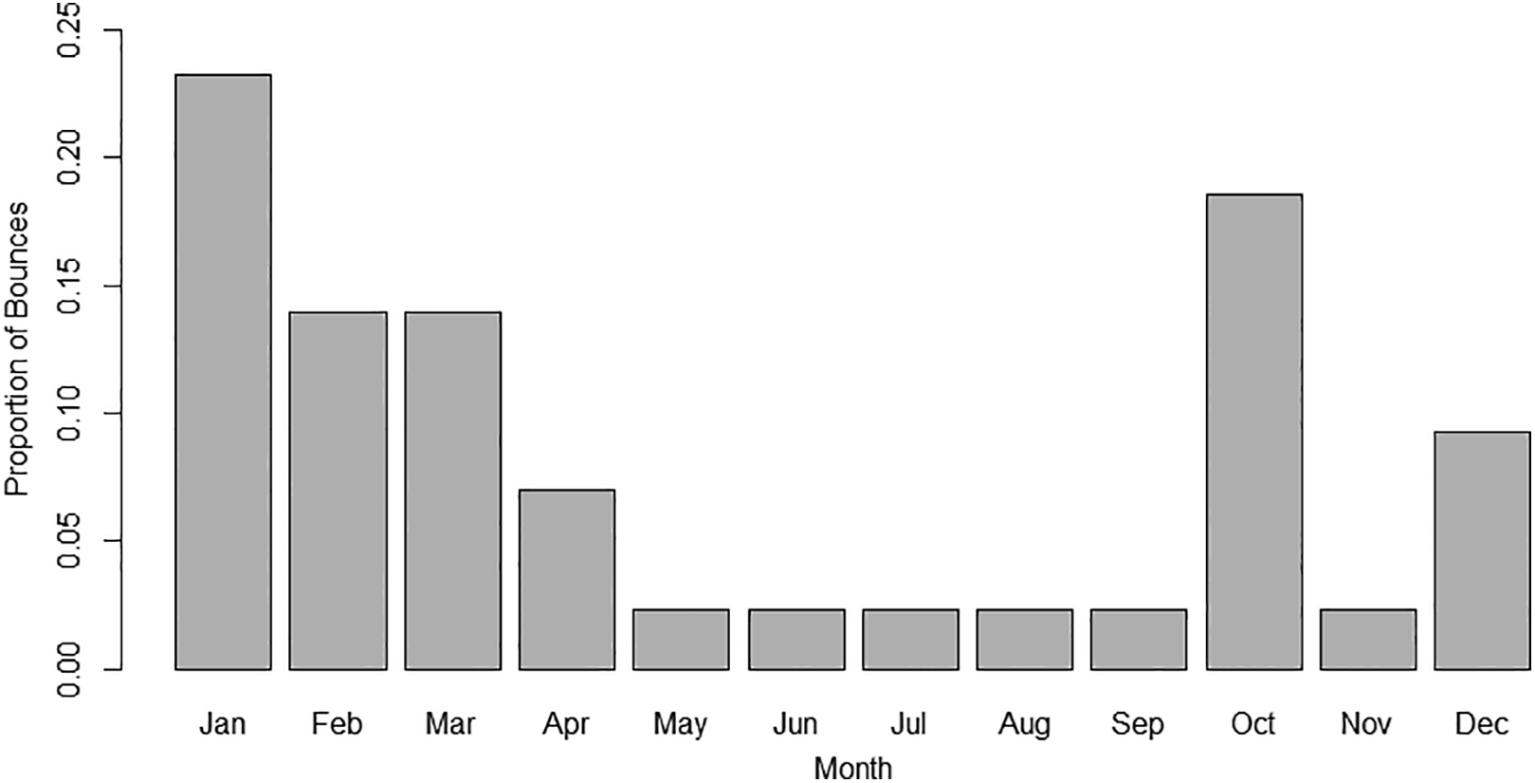
Proportion of bounces between home range segments (*n* = 44 bounces) by month for GPS collared bucks during 2017–2021 in central Mississippi, USA.

Frequencies of bounces between home range segments were not uniformly distributed across months [χ^2^ (11, *n* = 43) = 31, *p* ≤ .001] and peaked in January with 23% of all bounces followed by October with 19% (Figure 7). Bounces were generally centered around the breeding season and early springtime, with the exception of the pre‐breeding season spike in October. Bucks displaying bouncing behavior averaged 3.9 ± 1.6 bounces per year.

**FIGURE 8:**
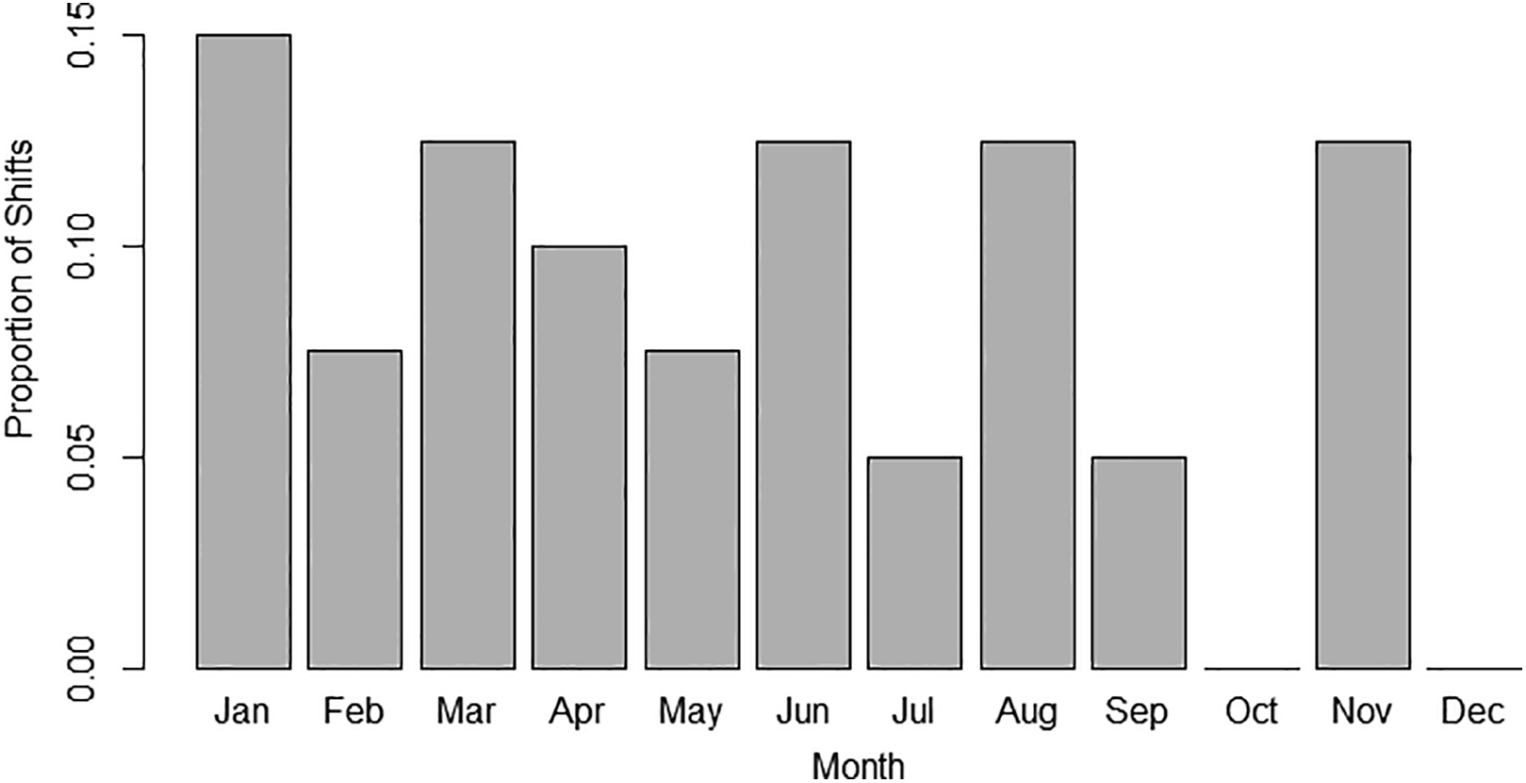
Proportion of shifts between home ranges (*n* = 40 shifts) by month for GPS collared bucks with a mobile strategy during 2017–2021 in central Mississippi, USA.

Mobile bucks shifted between home range segments a mean of 2.9 ± 1.5 times annually. Days spent per segment before shifting to another averaged 77.5 ± 62.4 for mobile bucks. Although there were subtle variations from month to month (Figure 8), no apparent pattern emerged for shift timing [χ^2^ (11, *n* = 40) = 13.4, *p* = .268]. Mean distance between 50% KDE core area centroids for mobile bucks was 7.1 km ± 7.1.

**FIGURE 9:**
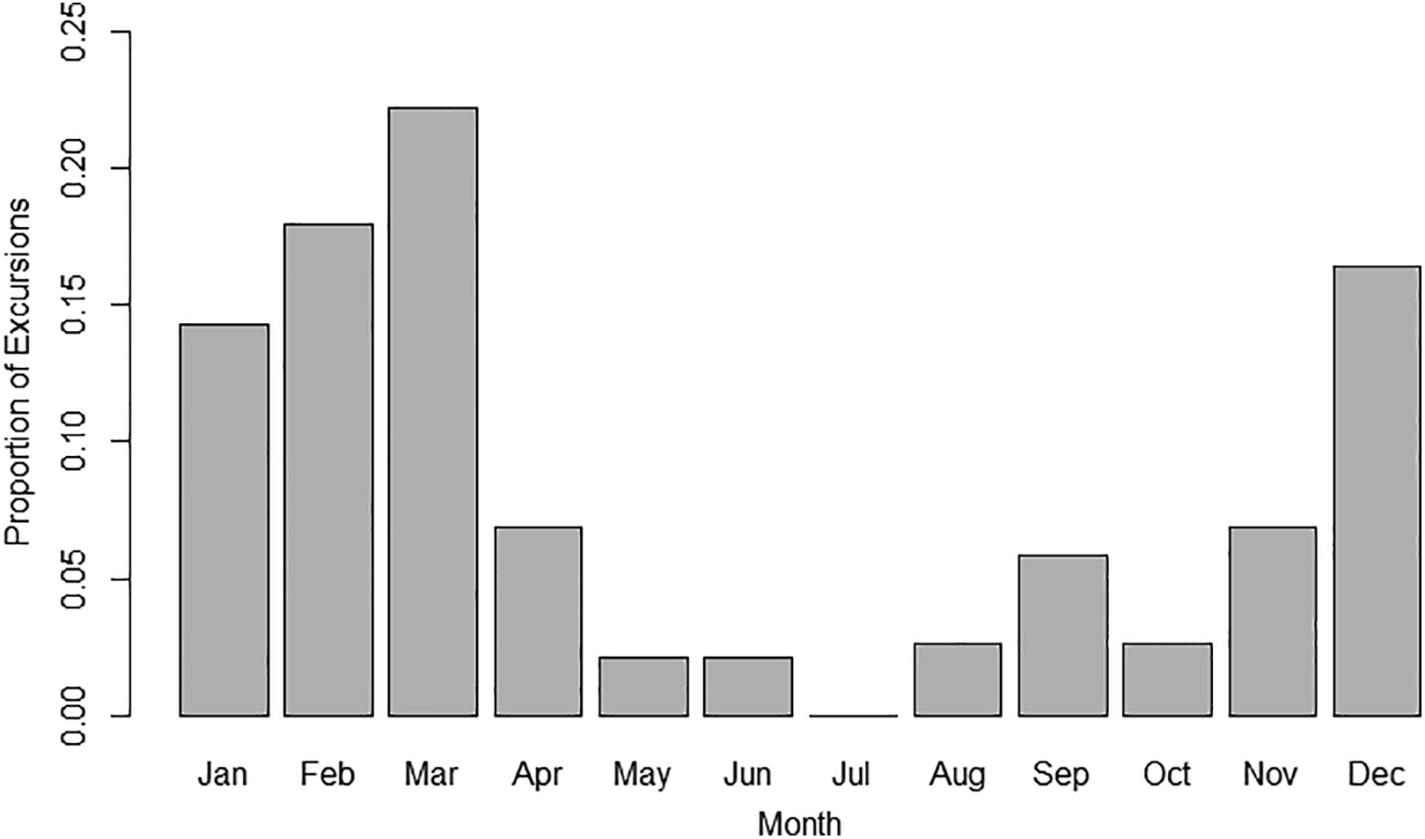
Proportion of excursions (*n* = 189 excursions) by month for GPS collared bucks during 2017–2021 in central Mississippi, USA. Excursions are any GPS point(s) ≥0.5 km outside a buck's 95% KDE isopleth.

We saw a strong strategy effect for a number of excursions, where sedentary bucks went on 5.9 annually (*F*_1,42_ = 16.62, *p* ≤ .001) while mobile bucks only went on 0.8 (*F*_1,42_ = 0.67, *p* = .42). However, when mobile bucks went on excursions, they lasted longer (mean = 25.5 h, *F*_1,187_ = 24.41, *p* ≤ .001) than sedentary buck excursions (mean = 12.7 h, *F*_1,187_ = 5.06, *p* = .03). Excursion distance for mobile (mean = 1.5 km) and sedentary bucks (mean = 1.1 km, *F*_1,187_ = 3.86, *p* = .06) did not differ. Excursion timing did not differ between sedentary and mobile bucks [*t*(11) ≤ 0.001, *p* = 1.0], so excursions for both classifications are combined in Figure 9. Excursion frequency was not uniform over time [χ^2^ (11, *n* = 189) = 138.05, *p* ≤ .001] and resembled bounce timing in that excursions peak in the breeding season through early spring and are infrequent from late spring through late summer.

We found no relationship between buck age and strategy (*F*_1,42_ = 0.60, *p* = .55), and no bucks switched strategies between years.

## 5 DISCUSSION

Our results provide a description of partial migration in a novel context void of chronic and severe environmental fluctuations. We found strong support for the hypothesis that deer populations in central Mississippi are partially migratory (Bocci et al., 2010). Despite unapparent environmental pressures that drive migratory behavior in higher‐latitude deer populations (Sabine et al., 2002), 33% of the bucks in our sample displayed such behavior. The resident (“sedentary”) strategy identified in the remaining 67% of bucks is the characteristic movement strategy reported for male and female deer in studies at similar latitudes (Karns et al., 2011; Thayer et al., 2009; Webb et al., 2010), and is the expected movement strategy for deer throughout the region where our study occurred given our bioclimatic conditions. Our study provides the first evidence that partial migration may apply to a larger proportion of lower‐latitude deer populations than originally thought (Mott, 1981), though the environmental justification for this partial migration is not clear.

The extreme fluctuations in seasonal resource availability inherent to high latitudes that drive migratory behavior in deer (Lesage et al., 2000; Sabine et al., 2002) and other species (Bocci et al., 2010; Somveille et al., 2015) are often not apparent at lower latitudes. However, a variety of environmental factors may influence the mobile strategy on our study sites. Deer occupying systems prone to extreme seasonal flooding are known to modify space‐use to avoid high water (Abernathy et al., 2019), but mobile bucks commonly shifted home range segments at times outside flooding events and several were never in proximity of a flooded river. Deer at latitudes characterized by long growing seasons with hot dry summers are commonly limited in terms of summer forage quality when nutritional demands are highest (Nichols et al., 2021), so seeking forage resources in multiple home range segments could be a plausible hypothesis for the mobile strategy. However, many sedentary bucks' home ranges overlapped those of mobile bucks and there were instances in which a mobile buck left an area only for the area to be occupied days later by another mobile buck, though it is possible for both mobile bucks to improve their nutritional availability relative to their previous segment in this circumstance. Research has shown predation risk influences deer movement strategies and space use (Little et al., 2014). Human hunters are the largest source of mortality for deer on our study areas (Henderson et al., 2020), and while it is impossible to rule out predation risk from predators such as bobcats (*Lynx rufus*) and coyotes (*Canis latrans*) driving the mobile strategy, the high proportion of shift dates outside of the hunting season make risk an unlikely sole driver. Individual environmental pressures could play a small role, but logic suggests none are solely responsible for the mobile strategy.

Likewise, biological factors might also influence the partial migration in lower‐latitude deer populations. An intriguing hypothesis is the mobile strategy is a vestigial behavior from migratory deer translocated to the region from higher latitudes during the restocking era. Deer from across the northern and eastern U.S. were translocated to Mississippi as part of extensive restocking efforts in the mid‐late 1900s (Deyoung et al., 2003), but past research indicates genetic signatures from source populations exist at low rates in the southern U.S. (Chafin et al., 2021; Deyoung et al., 2003; Youngmann, 2018) making the hypothesis unlikely. Further, although spatially heterogeneous breeding opportunities are hypothesized to drive movement strategies of adult bucks (Foley et al., 2015), they are unlikely the sole driver of the mobile strategy because shifts occurred at all times of year and were not constrained around the breeding season. We would also expect all mobile bucks to take full advantage of multiple home range segments in search of estrus females during the breeding season if this were the sole driver (Foley et al., 2018). Although a majority of mobile bucks displayed bouncing behaviors (70%), we would expect all mobile bucks to bounce between home range segments in proximity to the breeding season if heterogeneous breeding opportunities were the culprit. Another plausible argument is that young bucks assume the mobile strategy before becoming sedentary later in life. Bucks tend to decrease annual home range size as they age (Webb et al., 2007), and a strategy shift from mobile to sedentary could facilitate a similar result. Although our data indicate there is no relationship between age and strategy, a correlative relationship might emerge if we had a larger sample size or if individuals were evenly distributed across a wider range of ages. Last, interspecific or intraspecific competition may impact movement strategy. Deer‐deer competition for resources including cover, forage, and breeding opportunities could displace mobile bucks to new areas (Kjellander et al., 2004; Mysterud et al., 2011) and deer‐wild pig (*Sus scrofa*) competition for cover and forage could do the same in areas where wild pig abundance is high enough to restrict deer access to resources (McDonough et al., 2022). Although a possible explanation, our study design cannot identify competition as a driver of strategy. The disconnection between a single factor and the mobile strategy does not dismiss the notion that the cumulative effect of many factors could be responsible for partial migration in our study.

Identifying the source of strategy variation in our study is difficult, but the bouncing is clearly associated with the breeding season. Bouncing rates peak in late January, just days after peak breeding, and we hypothesize that bouncer bucks tend to remain in a home range segment while the majority of females are entering estrus before deeming it worthwhile to travel to their other segment in search of the last few receptive females. Similar timing and biological justifications are reported elsewhere for excursions and other movements (Debeffe et al., 2014; Karns et al., 2011). We suggest the secondary bounce peak in October may be a mechanism for bucks to locate groups of females in their other home range segment prior to the breeding season so they do not waste as much time searching when females are receptive (Foley et al., 2015). Although bucks lacking bouncing movements have the same opportunity to exploit breeding opportunities in multiple home range segments, they do not appear to take advantage of it. These bucks are either balancing the cost of increased energy expenditure and risk (predation, vehicle collisions, etc.) associated with inter‐segment travel by searching for mates in a single home range segment or they have not learned to seek mates in their other home range segment as it appears bouncer bucks have.

The timing of these long distance movements relative to the breeding season resembles reports from other studies (Karns et al., 2011; Kolodzinski et al., 2010), but breeding and springtime bounces and excursions appear to blend together rather than forming distinct movement peaks in breeding season and spring. This is likely because the breeding season in our study areas is approximately two months later than typical at higher latitudes, while spring green‐up occurs earlier. Shorter time between breeding season and spring green‐up likely causes long distance movements to blend together. Still, the high frequency of springtime bounces and excursions is additional evidence that these long‐distance movements are not restricted to breeding activities alone (Olson et al., 2015). Our results indicate sedentary bucks make more frequent excursions than mobile bucks. A possible explanation is that for mobile bucks, a resource may not be present in one home range segment but is seasonally predictable in another, while for sedentary bucks, the resource may not be as spatiotemporally predictable. This could lead to sedentary bucks making more short‐duration excursions in search of the unpredictable resource while mobile bucks have found and can rely on a more predictable resource patch, simply necessitating travel to the other home range segment to procure it, be it an area with known doe groups, a consistent nutritional source, or another resource (Mueller & Fagan, 2008; Roshier et al., 2008).

The most plausible driver of the mobile strategy may simply be its long‐term adaptive significance. Deer are characterized as colonizers that chase and rely on natural disturbances (McCullough, 1980), and these long‐distance movements may help them find new resources that confer survival advantages. Varied intraspecific movement behaviors likely convey differential strategies to exploit reproductive, forage, and/or cover resources (Garant et al., 2003; Harrington, 1961; Wheat et al., 2017). Since the minority (33%) of bucks in our sample displayed the alternative mobile strategy, the fitness‐level costs may be generally higher than for sedentary bucks while paying significant but less frequent fitness dividends. If this long‐term adaptive benefit is the driver of the mobile strategy in our populations, we find it peculiar that it has not been reported in other studies. The benefit of such long‐distance movements should extend beyond our study sites and be region wide, if not observable across the species' entire range.

Animals balancing a limited energy budget with survival and reproductive demands should make long‐distance movements when the benefit outweighs the cost. The high proportion of range shifts, bounces, and excursions we observed in spring correspond with increasing biomass of high‐quality forage resources (Merkle et al., 2016; Monteith et al., 2011). The elevated nutritional plane during spring green‐up may facilitate a higher proportion of long‐distance movements during springtime than in other seasons.

Although we separated bucks into discrete strategy categories based on home range characteristics for succinct analyses, strategies are, in reality, a continuum. Some sedentary bucks were quite mobile in terms of excursions, and some mobile bucks were quite sedentary, having small home range segments separated by just a few hundred meters. A strategy continuum of a population or a species likely conveys greater success in changing landscapes, where some individuals are better suited to a certain set of conditions than others (Chapman et al., 2011).

These movement strategies may benefit a deer population from a fitness perspective, but bucks with a mobile strategy introduce new complexity to disease management, specifically chronic wasting disease (CWD; Oyer et al., 2007). Not only must managers contend with humans moving deer across CWD management boundaries (Sigurdson & Aguzzi, 2007) and typical sedentary deer movements that transmit the disease at the intra‐population scale (Skuldt et al., 2008), but now they must consider the inter‐population transmission potential presented by mobile strategy deer. An individual male in our sample, buck #140, moved over 28 km straight‐line distance between centers of his home range segments, one of which is CWD positive and the other supposedly CWD negative at the time of his movements. Although the distance is comparable to long‐distance excursions reported elsewhere (Karns et al., 2011; Kilgo et al., 1996), mobile bucks present higher infection and transmission potential because they remain in home range segments for longer durations (77.5 days per segment vs. <25 h for excursions), thus increasing risk of CWD transmission between CWD positive and negative populations. Migratory movements, whether bounces or shifts, may increase the likelihood of disease transmission to naïve regions relative to populations displaying an exclusively sedentary strategy (Altizer et al., 2011). When present, these behaviors deserve special attention from managers and regulation‐setting agencies because of their unique and somewhat unexpected occurrence in southern deer populations.

This research also exposes a potential explanation for why some bucks vanish from a property around the same time each year (W. McKinley, personal communication, 13 April 2022). Many hunters share reports of individual bucks frequently detected on camera traps before disappearing for weeks or months at a time. Our data suggest these bucks are likely in another home range segment during long periods of absence. Such unconstrained movements should encourage proximate land owners to form cooperatives to develop large‐scale population management strategies (Mitterling et al., 2021) encompassing the movements of sedentary and mobile deer alike.

## AUTHOR CONTRIBUTIONS

**Luke Resop:** Formal analysis (equal); investigation (equal); methodology (equal); software (equal); visualization (equal); writing – original draft (equal); writing – review and editing (equal). **Stephen Demarais:** Conceptualization (equal); funding acquisition (equal); investigation (equal); methodology (equal); project administration (equal); supervision (equal); writing – review and editing (equal). **Bronson K. Strickland:** Conceptualization (equal); funding acquisition (equal); investigation (equal); methodology (equal); project administration (equal); supervision (equal); validation (equal); writing – review and editing (equal). **William T. McKinley:** Conceptualization (equal); investigation (equal); methodology (equal); project administration (equal); supervision (equal); writing – review and editing (equal). **Garrett Street:** Data curation (equal); formal analysis (equal); methodology (equal); software (equal); writing – review and editing (equal).

## FUNDING INFORMATION

Funding for this study was provided by Mississippi Department of Wildlife, Fisheries, and Parks.

## CONFLICT OF INTEREST STATEMENT

The authors declare that they have no known competing financial interests or personal relationships that could have appeared to influence the work reported in this paper.

## Data Availability

The data that support the findings of this study are openly available in Dryad at https://doi.org/10.5061/dryad.tdz08kq4w.

## APPENDIX 1

**TABLE A1:** Comparison of annual home range and other metrics used to differentiate, classify, and describe buck movement strategies for GPS‐collared bucks during 2017–2021 in central Mississippi, USA.

Metric	Sedentary	Mobile
50% KDE (ha)	70	1141
95% KDE (ha)	361	6530
50% Segmented KDE (ha)	NA	297
95% Segmented KDE (ha)	NA	2249
50% BBMM (ha)	66	97
95% BBMM (ha)	333	881
Distance between home range centroids (km)	NA	7.1
Days between shifts	NA	77.5
Pattern in excursion timing throughout year?	Peaks in breeding season through early springtime	
N excursions per year	5.9	0.8
Excursion distance (km)	1.1	1.5
Excursion duration (h)	12.7	25.5
Pattern in bounce timing throughout year?	NA	Peaks in Jan and Oct
N bounces per year	NA	3.9
Pattern in shift timing throughout year?	NA	Uniformly distributed
N shifts per year	NA	2.9

*Note*: The Kernel Density Estimation method (KDE) uses the ad‐hoc bandwidth estimator. The “Segmented KDE” method was applied only for mobile bucks, where spatially overlapping Lavielle segments were extracted, grouped, and run through KDE with the ad‐hoc bandwidth estimator. We also ran Brownian Bridge Movement Models (BBMM), using sig1 values estimated for each animal and a constant sig2 value of 20 m. Segmented KDE values were generated by extracting all relocations within a Lavielle segment, combining data from spatially overlapping segments, and estimating 95% and 50% KDEs on the segmented data, resulting in two separate sets of home ranges for each individual. Cells are merged where distinct values for each strategy are irrelevant or statistical tests revealed no difference between groups.
